# Genotypic variation in sorghum [*Sorghum bicolor* (L.) Moench] exotic germplasm collections for drought and disease tolerance

**DOI:** 10.1186/2193-1801-2-650

**Published:** 2013-12-04

**Authors:** Mohankumar H Kapanigowda, Ramasamy Perumal, Maduraimuthu Djanaguiraman, Robert M Aiken, Tesfaye Tesso, PV Vara Prasad, Christopher R Little

**Affiliations:** Ramasamy Perumal, Sorghum Breeder, Agricultural Research Center, Kansas State University, 1232 240th Avenue, Hays, Kansas 67601 USA; Department of Agronomy, Kansas State University, Manhattan, KS 66506 USA; Kansas State University, Northwest Research-Extension Center, Colby, KS 67701 USA; Department of Plant Pathology, Kansas State University, Manhattan, KS 66506 USA

**Keywords:** *Sorghum bicolor*, Climate change, Drought, Chlorophyll fluorescence, Charcoal rot, Fusarium stalk rot, Principle component analysis

## Abstract

Sorghum [*Sorghum bicolor* (L.) Moench] grain yield is severely affected by abiotic and biotic stresses during post-flowering stages, which has been aggravated by climate change. New parental lines having genes for various biotic and abiotic stress tolerances have the potential to mitigate this negative effect. Field studies were conducted under irrigated and dryland conditions with 128 exotic germplasm and 12 adapted lines to evaluate and identify potential sources for post-flowering drought tolerance and stalk and charcoal rot tolerances. The various physiological and disease related traits were recorded under irrigated and dryland conditions. Under dryland conditions, chlorophyll content (SPAD), grain yield and HI were decreased by 9, 44 and 16%, respectively, compared to irrigated conditions. Genotype RTx7000 and PI475432 had higher leaf temperature and grain yield, however, genotype PI570895 had lower leaf temperature and higher grain yield under dryland conditions. Increased grain yield and optimum leaf temperature was observed in PI510898, IS1212 and PI533946 compared to BTx642 (B35). However, IS14290, IS12945 and IS1219 had decreased grain yield and optimum leaf temperature under dryland conditions. Under irrigated conditions, stalk and charcoal rot disease severity was higher than under dryland conditions. Genotypes IS30562 and 1790E R had tolerance to both stalk rot and charcoal rot respectively and IS12706 was the most susceptible to both diseases. PI510898 showed combined tolerance to drought and Fusarium stalk rot under dryland conditions. The genotypes identified in this study are potential sources of drought and disease tolerance and will be used to develop better adaptable parental lines followed by high yielding hybrids.

**Electronic supplementary material:**

The online version of this article (doi:10.1186/2193-1801-2-650) contains supplementary material, which is available to authorized users.

## Introduction

By the end of the 21^st^ century, global surface temperatures are predicted to increase in the range of +1.4 to +5.8°C (IPCC 2007). Increased drought frequency is an important characteristic of predicted climate change and is the most important yield limiting abiotic stress worldwide (Araus et al. 2002; Meehl et al. 2007). Sorghum (*Sorghum bicolor* L. Moench) originated in Sub-Saharan Africa, and is a major food crop in arid and semi-arid regions of the world (Balota et al. 2008). Although sorghum has a wide range of adaptability and can be grown in a wide series of environments, including heat, drought, salinity and flooding (Ejeta and Knoll, 2007), this crop is usually affected by water stress at both pre- and post-flowering stages of development and has the most adverse effect on yield during and after anthesis (Tuinstra et al. 1997; Kebede et al. 2001; Blum 2004). Post-flowering drought stress reduces the number and size of the seeds per plant (Rosenow and Clark 1995) by 36 and 55%, respectively, which are the main causes for lower grain yield in sorghum (Assefa et al. 2010).

Due to its polygenic nature, drought tolerance is a complex trait. Drought is highly influenced by environmental conditions which makes breeding selection cycles difficult at pre- and post-flowering stages in sorghum (Tuinstra et al. 1996). However, progress in developing drought tolerant genotypes through breeding efforts is tremendous. Secondary component traits with moderate to high heritability values under drought stress include stay green, chlorophyll content, chlorophyll fluorescence, canopy temperature and transpiration efficiency (Harris et al. 2007; Kumar et al. 2008; Roháček et al. 2008; Liu et al. 2010; Kapanigowda 2011; Mutava et al. 2011; Talebi 2011). Improving these traits can increase drought tolerance potential in sorghum (Prasad et al. 2006; Borell et al. 2010). Kapanigowda (2011) observed high broad-sense heritability (0.77 to 0.90) for transpiration efficiency in sorghum and showed this trait could be improved through breeding. Mutava et al. (2011) studied diversity for chlorophyll content, leaf temperature, chlorophyll *a* fluorescence (PS II quantum yield), harvest index (HI) and yield among 300 genotypes from different races of sorghum and reported wide variability in sorghum.

Post-flowering drought stress is associated with charcoal rot and Fusarium stalk rot disease which leads to significant lodging and yield loss (Rosenow and Clark 1995; Tesso et al. 2004). However, the disease severity is high under hot and humid conditions (Tesso et al. 2012). Stalk rot is characterized by degradation of pith tissue near the base of the stalk as a result of senescence of the stalk pith cells (Tesso et al. 2012) resulting in reduced transportation of nutrients and water, and breakage of the stalk at the zone of infection causing lodging (Hundekar and Anahahosur 1994). Charcoal rot of sorghum is caused by *Macrophomina phaseolina* (Tassi) Goid. and is a serious problem under high soil temperature and low moisture by a prolonged dry period, particularly during the grain filling stage (Hassan et al. 1996). Fusarium stalk rot (caused by *Fusarium* spp.) is more severe when drought and high temperature stress occurs during grain development followed by wet, cool conditions near physiological maturity (Tesso et al. 2012). Several studies have been conducted to understand the influence of drought stress (Diourte et al. 1995; Seetharama et al. 1991; Tenkouano et al. 1993), nitrogen and plant growth (Cloud and Rupe 1994) and inheritance of resistance to *Fusarium* spp. and *M. phaseolina*. Seetharama et al. (1991) reported high incidence of charcoal rot and low grain yield in drought stressed sorghum plants. However, there was little attempt to identify genotypes in exotic germplasm collections that might serve as potential resistance sources to Fusarium stalk and charcoal rot for breeding programs. Even though drought and disease stresses commonly occur in sorghum production environments (Rosenow et al. 1996), there is little information on the interaction of drought on disease severity and drought tolerance in exotic sorghum germplasms. Of the total (44,773) accessions in the world sorghum germplasm collections, only 9,889 (22%) are photoperiod insensitive which are extensively used in breeding programs. The available genetic resources in sorghum are still under unexploited for diseases and drought stress tolerance (Rosenow and Dahlberg 2000). Thus, it is necessary to screen genotypes under both irrigated and dryland conditions to document environments that favor disease development. Evaluation of new germplasm based upon secondary traits that are associated with drought and disease tolerace is of paramount importance.. In the present study, 44 plant introductory (PI) and 84 minicore photoperiod insensitive germplasm accessions were evaluated along with 12 adapted lines. We hypothesized that there is genetic variation among the PI lines and sorghum minicore germplasm for post-flowering drought stress, *Fusarium* stalk rot (*F. thapsinum)* and charcoal rot (*M. phaseolina*) resistance. Identifying potential sources with post-flowering drought tolerance traits will combat disease development and result in increased grain yield. This study was conducted with the following objectives: (i) evaluate and identify sorghum exotic germplasm for drought, *Fusarium* stalk rot, and charcoal rot tolerance, and (ii) to identify traits confering tolerance to drought and stalk rots diseases.

## Materials and methods

### Genotypes and experimental design

A total of 245 minicore germplasm (10% from the 2247 core and 1% of the entire 37,000 world collections) were evaluated and identified in 2001 at ICRISAT (International Crops Research Institute for the Semi-Arid Tropics, Hyderabad, India) using hierarchical cluster analysis based on 11 qualitative and 10 quantitative traits (Upadhyaya et al. 2009). Of the 245 minicore germplasm, 84 photoperiod insensitive accessions with diverse origins and wide variability in flowering and plant height were used in this study. An additional 44 plant introductory (PI) exotic germplasm (fertility restoring R lines) of diverse origins were selected based on a preliminary drought stress field screening at the Agricultural Research Center, Hays, Kansas in 2008 and 2009 (data not presented) and 12 adapted B (male sterile maintainer) and R (fertility restorer) check lines: SC399B and BTx399 (parental lines used in hybrid seed production), RTx7078 (pre-flowering drought tolerant and post-flowering drought susceptible), SC599R (stalk rot resistant and post-flowering drought tolerant), AjabsidoR and KS19R (post-flowering drought tolerant), SC35R (charcoal rot resistant), 1790E R and BTx642 (stay-green), RTx7000 (susceptible to stalk rot and charcoal rot), BTx3042 (large seeded) and Laing Tang Ai R (high transpiration efficiency; Xin et al. 2009) were included in this study. The details of the genotypes used in this study are presented in Table 1.

**Table 1:** **List of exotic germplasm and adapted sorghum lines used in the study**

Entry*	Genotype	Origin	Entry	Genotype	Origin	Entry	Genotype	Origin
*Photoperiod insensitive minicore germplasm (84)*						*Other exotic germplasm (44)*		
1	IS608	USA	47	IS26737	South Africa	85	PI257309	Argentina
2	IS995	USA	48	IS26749	South Africa	86	PI295121	Australia
3	IS1212	China	49	IS28449	Yemen	87	PI236278	Australia
4	IS1219	China	50	IS28451	Yemen	88	PI510898	Botswana
5	IS1233	China	51	IS28614	Yemen	89	PI510920	Botswana
6	IS2205	India	52	IS29187	Swaziland	90	PI291382	China
7	IS2389	South Africa	53	IS29233	Swaziland	91	PI408822	China
8	IS2397	South Africa	54	IS29304	Swaziland	92	PI548007	China
9	IS2426	Afghanistan	55	IS29314	Swaziland	93	PI548034	China
10	IS2864	South Africa	56	IS29326	Swaziland	94	PI391652	China
11	IS2872	Egypt	57	IS29335	Swaziland	95	PI610730	China
12	IS3946	India	58	IS29358	Lesotho	96	PI276797	Ehiopia
13	IS3971	India	59	IS29468	Lesotho	97	PI576380	Ehiopia
14	IS4515	India	60	IS59519	Lesotho	98	PI262568	Fmr. Soviet Union
15	IS4631	India	61	IS29582	Lesotho	99	PI267109	Fmr. Soviet Union
16	IS4698	India	62	IS29627	South Africa	100	PI550685	Fmr. Soviet Union
17	IS5094	India	63	IS29654	China	101	PI585330	Hungary
18	IS8348	Pakistan	64	IS29689	Zimbabwe	102	PI586440	Hungary
19	IS8777	Uganda	65	IS29733	Zimbabwe	103	PI267392	India
20	IS12302	Zimbabwe	66	IS30231	Zimbabwe	104	PI267379	India
21	IS12706	USA	67	IS30383	China	105	PI533946	india
22	IS12735	Saudi Arabia	68	IS30507	Korea	106	PI562891	India
23	IS12804	Turkey	69	IS30508	Korea	107	PI536516	Maldives
24	IS12883	India	70	IS30533	Korea	108	PI264451	Spain
25	IS12945	Nicaragua	71	IS30536	Korea	109	PI534138	Sudan
26	IS13782	South Africa	72	IS30562	Korea	110	PI562166	Sudan
27	IS14010	South Africa	73	IS32295	India	111	PI550590	Ukraine
28	IS14090	Argentina	74	IS33844	India	112	PI534052	Uganda
29	IS14290	Botswana	75	IS473	India	113	PI591002	USA
30	IS17941	India	76	IS602	India	114	PI562723	USA
31	IS19389	Bangladesh	77	IS27912	South Africa	115	PI475432	Yemen
32	IS19445	Botswana	78	IS20743	India	116	PI533916	Zaire
33	IS19450	Botswana	79	IS20727	India	117	PI565174	Zimbabwe
34	IS20697	USA	80	IS19676	India	121	PI570959	Sudan
35	IS20816	USA	81	IS16151	India	122	PI570895	Sudan
36	IS21863	Syria	82	IS19262	Sudan	123	PI568992	Sudan
37	IS22294	Botswana	83	ICSR89058	India	124	PI 571032	Sudan
38	IS22616	Myanmar	84	IS4581	Yemen	125	PI 563146	Sudan
39	IS23992	Yemen				126	PI571165	Sudan
40	IS24348	India				127	PI569810	Sudan
41	IS24365	India				128	PI568323	Sudan
42	IS24453	South Africa				129	PI569809	Sudan
43	IS24463	South Africa				130	PI534052	Uganda
44	IS24492	South Africa				131	PI563253	Uganda
45	IS26694	South Africa						
46	IS26701	South Africa				*Adapted B and R check lines (12)*		
						118	BTx399	Texas, USA
						119	Laing TangAi R	China
						120	Ajabsido R	Sudan
						132	KS19R	Kansas, USA
						133	SC599R	Converted line
						134	1790E R	Texas, USA
						135	BTx642 (B35)	Texas, USA
						136	SC35R	Converted line
						137	SC399B	Converted line
						138	BTx3042	Texas, USA
						139	RTx7078	Texas, USA
						140	RTx7000	Texas, USA

**Figure 1:**
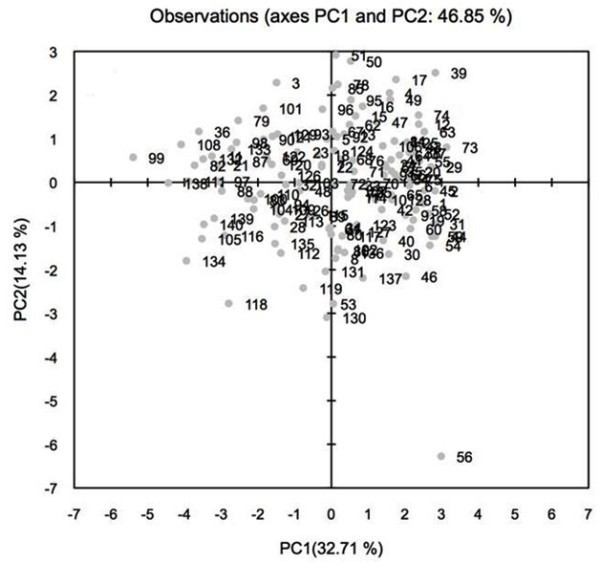
**Two dimensional plot of PC1 (principle component) versus PC2 for 140 sorghum genotypes segregating for drought related traits under both environments.** Note: List of 140 genotypes described in Table 1.

**Figure 2:**
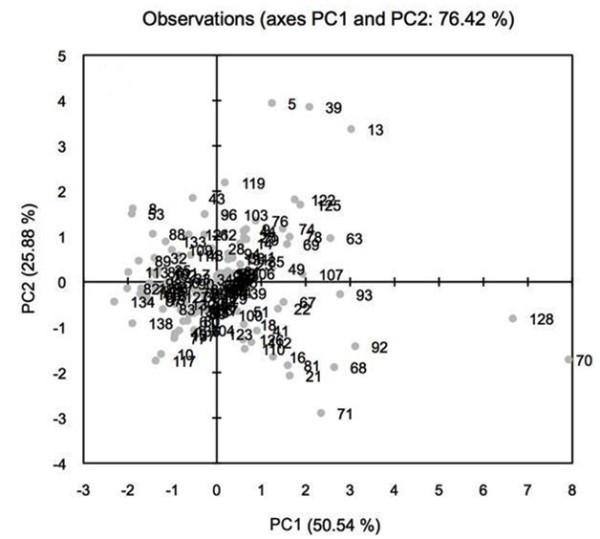
**Two dimensional plot of PC1 (principle component) versus PC2 for 140 sorghum genotypes segregating for stalk rot and charcoal rot lesion length.** Note: List of 140 genotypes described in Table 1.

**Figure 3:**
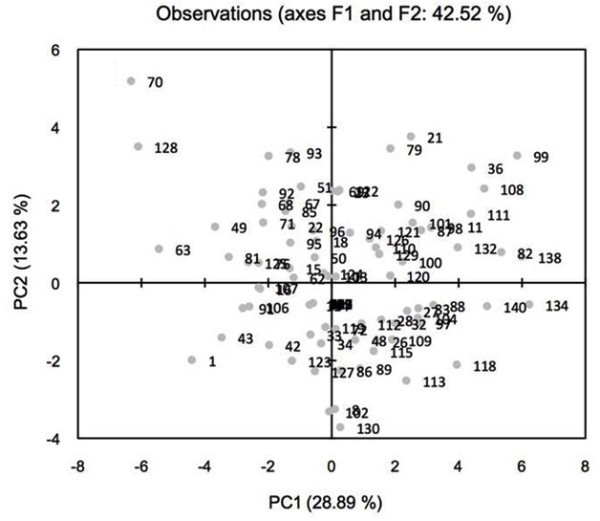
**Two dimensional plot of PC1 (principle component) versus PC2 for 140 sorghum genotypes segregating for combined drought and disease related traits.** Note: List of 140 genotypes described in Table 1.

*Entry numbers are used in Figures 1, 2 and 3.

Experiments were conducted at the Agricultural Research Center, Hays, Kansas (38.979°N latitude, 99.326°W longitude; 611 m elevation above sea level), under irrigated and dryland conditions in summer 2011. A total of 140 genotypes were sown in a randomized complete block design with two replications. The soil type at the experimental site was a Harney silt loam (fine smectitic mesic Typic Argiustoll) and was fertilized with 89.6 kg N ha^-1^ during planting. Seeds of each genotype were treated with fungicide (ethanethiol or ethyl mercaptan (Captan) @ 2 mL kg^-1^ seed) and were planted in single-row plots with 9.14 m length and 0.76 m width with 100 seeds per row.

### Irrigation and environmental conditions

Experiments under irrigated conditions were managed by providing additional irrigation of 35.0, 32.7 and 40.8 mm at 40 (14 July 2011), 52 (26 July 2011) and 86 (29 Aug 2011) days after planting (DAP), respectively through a skip furrow-row irrigation system. Irrigation was provided before the plants showed wilting symptoms (leaf rolling). The dryland experiment was rainfall dependant. The rainfall received was 276 mm during the crop growth period and it came within 34 days of cropping season. The number of rainy days (> 5 mm) received during the cropping season were 16 with 3, 3, 4, 1 and 5 days in June, July, Aug, Sep and Oct, respectively (Additional file 1: Figure S1). Therefore, the crop experienced drought stress during 80 to 110 DAP. In addition, there was a 19% decrease in total rainfall during the cropping season compared to the normal average over 30 years. The average air temperature during seedling and vegetative stages of the cropping season ranged from 25 to 29°C (Additional file 1: Figure S1), while the temperature during flowering and grain filling ranged from 18 to 27°C.

### Drought stress

Three plants per genotype in each replication, both in the irrigated and dryland experiments, were selected based on phenotypic uniformity namely flowering date, plant height and panicle exsertion. The plants were tagged to record PS (photosystem) II quantum yield (Fv/Fm), canopy temperature and chlorophyll content (SPAD). Chlorophyll content was measured at 59, 76 and 103 DAP; PS II quantum yield at 67 and 83 DAP; and leaf temperature at 61 and 81 DAP. Chlorophyll content was recorded on the flag leaf using the SPAD (Soil and Plant Analytical Development) meter (Minolta SPAD 502, Spectrum Technologies, Inc., Plainfield, Illinois, USA). Leaf temperature was measured from the flag leaf on each tagged plant using a forward-looking infrared (FLIR) camera (EXTECH i5, Extech Instruments Corp., Nashua, New Hampshire, USA) on clear, hot sunny days between 11.00 am and 2.00 pm. PS II quantum yield was measured from 30-min dark-adapted flag leaves (Prasad et al. 2008) using a pulse modulated, hand held, OS-30 p chlorophyll fluorometer (Opti-Sciences, Hudson, New Hampshire, USA). Plant height (cm) was measured from ground to the tip of the panicle on the main stem. Days to mid-anthesis was recorded as number of days from planting until 50% of the panicles were at mid-anthesis on a whole plot basis. At harvest, grain yield and biomass were obtained from each genotype by arbitrarily sampling three uniform plants and calculating harvest index (HI) using the procedure of Prasad et al. (2008).

### Disease stress

#### Inoculum preparation

Inoculum suspensions of *Fusarium thapsinum* (stalk rot) and *Macrophomina phaseolina* (charcoal rot) pure cultures were initiated in potato dextrose broth. The suspensions were incubated on a shaker at room temperature until conidia (*F. thapsinum*) or mycelium (*M. phaseolina*) was produced. *F. thapsinum* suspensions were strained through four layers of cheesecloth to separate conidia from mycelial masses. *M. phaseolina* did not produce conidia, therefore the mycelium was blended, and the suspension was strained through cheesecloth, as described for *F. thapsinum*, to separate small hyphal fragments. Concentrations of conidia or hyphal fragments were determined using a hemacytometer or counting chamber. The final concentration of both pathogens was adjusted to 2 × 10^5^ conidia or hyphal fragments mL^-1^ by diluting with 10 mM phosphate-buffered saline (pH 7.2).

#### Field inoculation and measurements

During anthesis, a total of nine plants per genotype in each replication in both dryland and irrigated experiments were tagged for artificial inoculation with *F. thapsinum*, *M. phaseolina*, and sterile water (control), respectively (*i.e.* three plants per treatment). Plants were inoculated at 10 days after flowering at the rate of 1 mL of inoculum per plant between the bottom-most node and brace node. Artificial inoculation was performed using a syringe and pre-wounding the plant with a fine-bit drill. Plants were evaluated for resistance to stalk and charcoal rot by measuring the lesion length by splitting the stem at 28 days post-inoculation (DPI) and measuring the spread of red discoloration along the length of the pith from the point of inoculation. Panicles were harvested from the same plants to compare the yield reduction (%) due to stalk and charcoal rot.

### Data analysis

Analysis of variance was carried out using the PROC GLM procedure of SAS (version 9.1.3) by evaluating genotype effects for each environment. A randomized complete block design was used with two replications. Means separation was carried out by environment for both drought and disease related traits using Fischer’s protected LSD (SAS, v 9.1.3). The data on chlorophyll content, leaf temperature and PS II quantum yield at various stages were averaged to get the main effects of genotype. Genotypes were grouped into tolerant and susceptible for each trait based on their mean performance across the environments. Combined analyses (both irrigated and dryland) were done to identify tolerant and susceptible genotypes for each trait. Relative performance of a genotype was calculated by comparing to the check genotype BTx642 for drought related traits. Variance components for genotype were estimated and used to calculate repeatability estimates to increase precision in selection based on repeatability (Knapp et al. 1987 and represents the proportion of total variance in multiple measurements of a trait that is due to differences among genotypes (Dohm 2002). Repeatability values were obtained by subtracting the fraction of the total phenotypic variance attributable to variance between repetitions (Falconer and Mackay 1996) using type III sum of squares in PROC GLM in SAS 9.1 (SAS Institute, Cary, NC). Principle component analysis (PCA) was performed to evaluate associations among the genotypes and among the variable using the XLStat (Addinsoft, Paris, France) software package.

## Results

### Genotype reaction to drought stress

Mean performance and analysis of variance of the genotypes for chlorophyll content, PS II quantum yield and leaf temperature for both environments averaged over different stages of observation and other agronomic traits are presented in Additional file 2: Tables S1 and S3 and were used to understand the reaction of the genotypes to drought stress as it showed significant differences among all genotypes for all the traits. The mean plant height among genotypes over two environments was 119 cm. Genotypes exhibited an average plant height of 175 and 223 cm under dryland and irrigated conditions, respectively (Table 2). Of the 140 genotypes, 105 exhibited mean plant heights ranging from 150 to 331 cm and the remaining were < 150 cm (Additional file 2: Table S1). Across environments, genotypes IS23992, IS28449, and PI408822 were the tallest (288 to 331 cm), and BTx399, SC299, and BTx3042 were the shortest (75 to 82 cm) (Additional file 2: Table S1). Days to flowering ranged from 61 to 105 days over two environments. Most of the exotic germplasm accessions were in the range of 82 to 106 days to 50% flowering. All adapted lines flowered between 61 and 75 DAP. Genotypes IS8777, IS26694 and IS14290 were late (100 to 106 days) and IS12804, IS12706 and PI291382 reached early 50% flowering (Additional file 2: Table S3).

**Table 2:** **Mean performance of agronomic and drought stress related physiological traits**

Environment	Plant height (cm)	Chlorophyll content (SPAD value)	PS II quantum yield	LT (°C)	Days to flowering	Grain yield (g plant^-1^)	HI
Dry land	175.3b*	44.59b	0.751b	37.06a	81.89a	20.79b	0.35b
	(60.10-297.51) †	(22.66-61.92)	(0.70-0.78)	(33.83 - 40.4)	(59.10-101.2)	(4.81-66.40)	(0.02-0.37)
Irrigated	222.2a	48.84a	0.759a	36.09b	81.53a	37.62a	0.42a
	(72.53-365.21)	(25.04-63.65)	(0.61-0.79)	(34.23-42.46)	(60.12-104.41)	(7.12-77.97)	(0.03-0.45)
LSD (0.05)	5.72	0.71	0.0045	0.58	0.91	0.88	0.1

SPAD = chlorophyll content; LT = leaf temperature; HI = harvest index; *Means followed by same letters in a column are not significantly different at LSD (*P* < 0.05); †Values in the parentheses indicate the mean range.

Drought stress in dryland conditions decreased chlorophyll content (SPAD value), grain yield and HI by 9, 44 and 17%, respectively, over irrigated conditions (Table 2). Based on the average of three measurements, genotypes PI264451, PI267109 and BTx3042 had increased chlorophyll content between 15 and 21% compared to the check BTx642. In contrast, genotypes SC 399, IS29654, IS1219 and IS995 had decreased (28 to 55%) chlorophyll content compared to BTx642 under dryland conditions. Similarly, the genotypes, IS28451, RTx7000 and BTx642 had increased PS II quantum yield (0.78 to 0.79) and IS2426, SC399 and IS1219 had decreased PS II quantum yield (0.67 to 0.71; Additional file 2: Table S3) under dryland conditions.

Under dryland conditions, genotypes RTx7000, PI562723, IS24453, PI475432, and IS2397 had increased leaf temperature (41 to 42°C) and Ajabsido, KS19, PI570895, IS29654 and IS26694 had decreased leaf temperature (34 to 36°C) (Table 3). The check (BTx642) had a leaf temperature of 40°C. However, there was not much variation in leaf temperature (35 to 37°C) among these genotypes under irrigated condition (Table 3). Under dryland conditions, genotypes with increased leaf temperature had a grain yield of 15 to 35 g plant^-1^. However, the genotypes with lower leaf temperatures had a grain yield of 7.7 to 43 g plant^-1^ (Table 3). The top five genotypes showed a 2.6 to 3.8% increase in leaf temperature, while the bottom five showed a 6 to 16% decrease in leaf temperature when compared to BTx642 (Additional file 2: Table S3). Genotypes PI510898, IS1212 and PI533946 had higher yields under dryland conditions which is 57, 38 and 38% increase over the check BTx642. Similarly, genotypes PI408822, Ajabsido and IS26737 had high yields under irrigated conditions (Additional file 2: Table S3). Mean HI was high (0.42) in irrigated when compared to dryland (0.35) conditions (Table 2). Genotypes PI475432, IS3971 and IS30562 exhibited high (0.48 to 0.57) and IS26694, IS14290 and IS12945 exhibited low (<0.1) HI under dryland conditions (Additional file 2: Table S3) when compared to other genotypes. Genotypes PI570959, PI565174, IS17941 and IS12883 showed a 6 to 52% decrease in HI when compared to BTx642 under the dryland environment.

**Table 3:** **Mean performance of genotypes based on leaf temperature (LT), grain yield and harvest index (HI) under dryland and irrigated environments**

Dryland environment				Irrigated environment		
Genotype	LT (°C)	Grain yield (g plant^-1^)	HI	LT (°C)	Grain yield (g plant^-1^)	HI
**Top five genotypes**						
RTx7000	42.3	30.4	0.33	35.4	40.1	0.36
PI562723	42.3	22.3	0.34	35.2	14.6	0.40
IS24453	41.5	15.0	0.19	36.8	49.2	0.13
PI475432	41.5	34.4	0.37	35.1	39.0	0.16
IS2397	41.4	15.5	0.18	36.6	42.2	0.30
**Bottom five genotypes**						
Ajabsido	34.8	27.2	0.37	36.1	71.1	0.29
KS19R	35.8	24.3	0.44	36.6	55.7	0.31
PI570895	36.3	43.3	0.40	36.0	55.3	0.26
IS29654	36.4	7.7	0.16	37.2	1.16	0.04
IS26694	36.4	12.0	0.10	36.5	45.0	0.13

### PCA for drought related traits

PC1 separated the genotypes based on plant height and days to flowering. PC2 separated the genotypes based on chlorophyll content, PS II quantum yield, leaf temperature and HI (data not shown). The tallest (IS23992, IS5094, IS28451 and IS28614) and the shortest (BTx399 and 1790E) genotypes were differentiated by the PC1 loading value. A clear differentiation was observed between genotypes (PI267109, PI264451 and BTx3042) with increased and decreased (IS29326) chlorophyll content and HI. The PC2 loading value differentiated the genotypes with increased PSII quantum yield (IS28451, IS23992 and IS28614) and PC1 loading value with decreased PSII quantum value and delayed flowering (IS29326 and IS2670) (Figure 1).

### Genotype reaction to Fusarium stalk rot and Charcoal rot inoculation

Significant effects of genotypes were observed for Fusarium stalk rot and charcoal rot lesion length under both environments (Additional file 2: Table S2). Mean lesion length among the genotypes for both pathogens inoculations were 8.4 and 9.3 cm in dryland and 10.4 and 13.0 cm in irrigated environments, respectively (Table 4).

**Table 4:** **Mean performance of Fusarium stalk rot and charcoal rot on lesion length and grain yield in sorghum exotic germplasm and adapted lines**

Environment	Lesion length (cm)			Grain yield (g plant^-1^)		
	***F. thapsinum***	***M. phaseolina***	***Control***	***F. thapsinum***	***M. phaseolina***	Control
Dryland	8.44b*(0.11-38.10)	9.39b (0.10-35.58)	2.90b (0.11-4.55)	31.52b (0.85-72.55)	35.73b (6.91-78.86)	33. 55b (0.23-79.15)
Irrigated	10.47a (0.12-40.50)	13.01a (0.11-64.10)	4.67a (0.02-5.56)	42.06a (2.43-79.20)	43.94a (5.25-84.89)	40.47a(1.25-87.47)
LSD (0.05)	0.92	0.86	0.93	2.97	3.51	2.87

*Means followed by same letters are not significantly different according to LSD (*P* < 0.05) within trait and between environments; Values in parentheses indicate the mean range.

Under dryland conditions, genotypes IS30562, IS14010 and PI510920R had lower lesion length of (2.7 to 4.5 cm) for Fusarium stalk rot with grain yield ranging from 31.4 to 37.3 g plant^-1^. However, the genotypes IS30536, IS29654, and IS12706 had lesion lengths of 14.0 to 29.2 cm with grain yield ranging from 7.6 to 14.0 g plant^-1^ (Table 5). Similarly, the top three genotypes were with lower lesion length (2.8 to 3.0 cm) for *M. phaseolina* with a grain yield ranging from 16.4 to 47.7 g plant^-1^. The genotypes with increased lesion length (17.4 to 38.0 cm) had a grain yield ranging from 18.3 to 28.9 g plant^-1^. It is obvious from the present study that there are genotypes with lower lesion lengths and higher grain yield (Table 5).

**Table 5:** **Mean performance of genotypes (top and bottom) for lesion length and grain yield in response to Fusarium stalk and charcoal rot under the dryland environment**

Genotype	***F. thapsinum***			Genotype	***M. phaseolina***		
	Lesion length (cm)	***F. thapsinum*** grain yield (g plant^-1^)	Control grain yield (g plant^-1^)		Lesion length (cm)	***M. phaseolina*** grain yield (g plant^-1^)	Control grain yield (g plant^-1^)
**Top five genotypes**							
IS30562	4.50	37.30	47.45	1790E R	2.77	16.43	20.36
IS14010	4.42	34.98	46.45	IS 26749	3.00	37.75	49.25
PI510920R	2.75	31.43	35.73	BTx399	3.03	47.73	55.20
BTx399	6.92	30.28	55.20	IS19445	3.58	29.1	38.25
PI510898R	3.50	29.45	58.65	IS24463	4.33	39.98	42.95
**Bottom five genotypes**							
IS30536	29.17	7.60	17.33	PI568323R	38.00	26.00	28.85
IS29654	14.00	10.45	27.75	PI548034R	28.25	18.30	46.85
IS12706	19.20	14.04	34.14	IS12706	17.42	25.71	34.14
PI548007R	18.67	15.25	28.89	IS16151	14.92	28.88	30.75
RTx7078	12.30	25.01	30.29	PI391652R	10.97	25.19	48.88

Under irrigated conditions, it was observed that different sets of genotypes were found to be tolerant and susceptible for both pathogens when compared to dryland conditions (Table 6). The control plants (water inoculated) had higher grain yield than plants inoculated with either pathogen. Genotypes IS59519, PI562166R and IS14090 had the lowest lesion length (*F. thapsinum* inoculated) of 5.9 to 7.3 cm with a grain yield ranging from 38.6 to 54.3 g plant^-1^ when compared to genotypes IS30533, IS608 and IS22616 having the highest lesion length of 18.1 to 28.3 cm with the lowest grain yield ranging from 12.8 to 24.1 g plant^-1^ (Table 6). Similarly, genotypes KS19R, IS22294 and IS12706 had the lowest lesion length (*M. phaseolina* inoculated) of 2.5 to 6.5 cm with the highest grain yield ranging from 35.1 to 42.1 g plant^-1^ when compared to genotypes IS33844, PI570895R and PI548007R, which had the highest lesion length of 15.5 to 33.0 cm, with the lowest grain yield ranging from 15.3 to 28.9 g plant^-1^ (Table 6).

**Table 6:** **Mean performance of genotypes (top and bottom) for lesion length and grain yield in response to Fusarium stalk rot and charcoal rot under the irrigated environment**

Genotype	***F. thapsinum***			Genotype	***M. phaseolina***		
	Lesion length (cm)	***F. thapsinum*** Grain yield (g plant^-1^)	Control Grain yield (g plant^-1^)		Lesion length (cm)	***M. phaseolina*** Grain yield (g plant^-1^)	Control Grain yield (g plant^-1^)
**Top five genotypes**							
IS59519	7.25	54.33	64.20	KS19R	2.50	42.13	42.43
PI562166R	7.00	44.13	69.05	IS22294	6.50	35.90	66.05
IS14090	5.88	38.60	49.08	IS12706	6.25	35.15	40.62
PI576380R	6.50	37.33	44.78	IS23992	6.00	33.85	68.25
IS29582	8.13	35.97	27.64	PI267379R	5.75	30.98	41.08
**Bottom five genotypes**							
IS30533	28.25	12.78	61.98	IS33844	33.00	15.35	62.85
IS608	18.13	22.18	34.10	PI570895R	32.88	26.05	49.38
IS22616	18.50	24.13	36.10	PI548007R	17.00	28.90	47.00
Laing TangAi R	21.50	26.05	32.10	IS20743	19.00	16.30	18.04
PI548034R	25.50	31.30	83.05	IS608	15.50	23.25	34.10

### PCA for disease related traits

Disease related traits revealed 76.3% of the variability explained by the first two PCA components. PC1 and PC2 respectively accounted 50.5 and 25.8% of the total variability for lesion length (Figure 2). Component loading revealed the highest positive values of PC1 with increased lesion length due to *F. thapsinum* and *M. phaseolina* inoculation indicating susceptibility to these pathogens (data not shown). Lower negative PC1 and positive PC2 values represent shorter lesion length and indicate genotypes with tolerance to Fusarium stalk rot and charcoal rot.

## Discussion

The present study showed wide genetic variability among the sorghum genotypes for drought and disease tolerance, which provides more opportunities for effective selection. Post-flowering drought stress significantly increased chlorophyll loss and decreased PS II quantum yield in all the genotypes (Table 2). The reduction in chlorophyll content and PS II quantum yield activities under drought stress could be due to thylakoid membrane damage caused by increased production of reactive oxygen species (Tambussia et al. 2000). Enzymes involved in chlorophyll biosynthesis are bound to membranes (Tewai and Tripathy 1998); and dilation of thylakoids by drought stress can also cause chlorophyll loss (Bukhov et al. 1999). Drought stress decreased PS II quantum yield indicating increased permeability of thylakoid membranes resulting in proton leakage across the thylakoid membrane which decreases ATP and NADPH production and decreased photochemical efficiency (Schrader et al. 2004). Miyashita et al. (2005) found that PS II quantum yield level was decreased in kidney beans (*Phaseolus vulgaris* L.) due to drought stress, suggesting reduction in electron transfer to photosystem II. Mutava et al. (2011) observed similar results in grain sorghum genotypes. In the present study, genotypes PI264451 and PI267109 had increased chlorophyll content, and IS28451 and RTx7000 had increased PS II quantum yield under dryland condition (Additional file 2: Table S1). The above-mentioned genotypes can be used in drought tolerance breeding for introgression of the stay green trait.

Seversike et al. (2012) showed that air temperature altered transpiration responses to vapor pressure deficit (VPD). Controlled transpiration rate under moisture limited condition shows drought tolerance in crop plants. Under environments where VPD is high, genotypes having limited transpiration rate may yield more due to significant water savings. Leaf temperature is positively correlated with transpiration rate and can be of a surrogate measure for drought tolerance. Our data showed that genotypes RTx7000 and PI475432 had increased leaf temperature (42 to 41°C) in the dryland environment with slightly higher grain yield (30 to 34 g plant^-1^) relative to the other genotypes. Since, RTx7000 is a non-stay green-genotype, it contrasts with the finding of Jordan et al. (2012) that the stay-green trait may result from decreased water use early in the season, allowing water to be conserved to sustain a longer period of grain filling. However, it confirms to the finding of Choudharya et al. (2013) that genotypes expressing the breakpoint in transpiration rate with increasing VPD were found among both stay-green and non-stay-green genotypes. However, the genotype PI570895 had a leaf temperature of 36°C and grain yield of 43 g plant^-1^ (Additional file 2: Table S3) indicating that this genotype may have availed more soil moisture and cooled the canopy favors higher grain yield (Table 3). This type of genetic variability was also observed by Mutava et al. (2011) on sorghum. Similar extreme variation was observed by Gholipoor et al. (2010) among sorghum genotypes in transpiration response to vapor pressure deficit (VPD). They identified seventeen sorghum genotypes exhibiting a breakpoint in their VPD response in the range from 1.6 to 2.7 kPa, above which there was little or no increase in transpiration and concluded the possibility of soil water conservation upon utilizing these genotypes. Overall, considering leaf temperature, chlorophyll content, and PS II quantum yield, genotypes PI510898, IS1212, PI533946, PI550590, IS2872 and PI562166 may have drought tolerance ability as evidenced by higher grain yield under dryland conditions (Additional file 2: Table S3). Repeatability estimates for SPAD, PS II quantum yield, plant height, days to flowering, lesion length for Fusarium stalk rot, charcoal rot and grain yield were moderate to higher for both environments (Additional file 2: Tables S1 and S2). These repeatability estimates indicated that these traits can be improved through selection as this tool quantifying the extent to which one trait’s performance remains consistent over environments.

Growing environment significantly affected Fusarium stalk rot. The weather during the cropping season was characterized by intermittent rainfall and high temperature stress early in the season. However, later in the season wet and cool weather was followed by dry conditions (Additional file 1: Figure S1), which favors disease development (Tesso et al. 2012). Under dryland condition, moisture stress later in the season was associated with significantly lower lesion length compared to the irrigated environment (Table 4). These results were consistent with the results reported earlier by Tesso et al. (2004), which showed that lower mean disease score was due to lack of adequate late season moisture in sorghum. Significant differences in mean lesion length were noted between genotypes (Table 4). These differences are assumed to reflect differences in disease susceptibility of the genotypes through mobilization of stalk carbohydrate reserves to developing seed during grain development (Tesso et al. 2012) rather than differences in host defense responses. Because of lost reserves, the stalk tissue might become senescent earlier to favor stalk and charcoal rot infection. Lower lesion lengths for both stalk rot and charcoal rot inoculation were observed in the genotype IS30562 and 1790E R, respectively under the dryland environment. The common susceptible sources for both diseases were IS12706 with high mean lesion length under the dryland condition (Table 5). Similarly, IS59519 and PI562166R were tolerant to Fusarium stalk rot and KS19R and IS22294 were tolerant to *M. phaseolina* rot under irrigated conditions (Table 6). The genotypes IS30533 and IS33844 were found to be susceptible to Fusarium stalk rot and charcoal rot under irrigated conditions (Table 6).

BTx399, a stay-green line showed a high degree of tolerance to *M. phaseolina*, with low lesion length relative to other adapted lines, (Tuinstra et al. 1996; Xu et al. 2000; Kebede et al. 2001). This line was also drought tolerant and Fusarium stalk rot resistant (Tao et al. 1993). This may be due to the retention of a higher proportion of assimilates in the stem by stay green lines later in the growing season (Seetharama et al. 1991). Tesso et al. (2004) reported that SC599 shows high level of resistance to *F. proliferatum*, and it can be used as a potential source of resistance to both *Fusarium* stalk rot disease and charcoal rot. Tenkouano et al. (1993) also reported that B35, which is a close relative of SC35 and SC599 and their hybrids showed high levels of resistance to *M. phaseolina*. These results indicate that stalk resistance in SC599 and B35 are different and cannot be equated (Tenkouano et al. 1993). The simplest explanation for these differences is that different genes are involved in regulating stalk rot resistance in these two genetic backgrounds (Tesso et al. 2005). Similarly, BTx399 may also have a different genetic background, which governs the resistance to Fusarium stalk rot and charcoal rot.

PCA on drought related traits revealed that PC1and PC2 accounted 34.5% and 14.3% of the total variability respectively. Further, PCA revealed a significant negative correlation between days to flowering and HI. This makes sense since late flowering results in a shorter grain filling period in sorghum, where the duration of the reproductive stage is fixed (Barnabas et al. 2008). In this study, plant height exhibited significant positive correlation with days to flowering (*r* = 0.37, *P* < 0.0001) and negative correlation with harvest index (*r* = −0.56, *P* < 0.0001). A similar relationship was observed in earlier studies by Murray et al. (2008), Ritter et al. (2008) and Zhao et al. (2009) in sorghum. These authors concluded that taller sorghums have the advantages of accumulating more biomass due to greater translocation of photosynthates from the vegetative tissues resulting in late maturity and low grain yield. Also, some tall sorghums may be prone to lodging, which results in low harvest index (Rooney 2004; Murray et al. 2009). Genotypes of this group may utilize the available soil water for vegetative development, leaving no moisture for the grain filling stage concomitant with lower current photosynthesis during post-flowering stages and decreased grain yield. A significant positive association was observed between chlorophyll content under dryland and HI under both environments. Richards (2000) suggested that increased photosynthetic rate through improved leaf chlorophyll content can improve grain productivity and HI of the genotype. Leaf temperature had lower negative values of PC2 in both environments and chlorophyll content and PS II quantum yield had positive values. This clearly indicates that genotypes with extreme leaf temperatures (>35°C) might experience damage to leaf photosynthetic apparatus and chlorophyll loss (Djanaguiraman et al. 2011).

PCA on combined drought and disease related traits showed positive correlation between PS II quantum yield, chlorophyll content and HI indicating these traits are associated with drought and disease tolerance (Nazir Mir et al. 1998). However, these have to be verified using proven disease tolerant and susceptible genotypes under controlled and field experiments. Plant height was positively correlated with lesion length indicating that disease development is directly proportional to plant height which was also confirmed by component loading value (Bazzalo et al. 1991). Genotypes PI267109, IS12706, PI533916, BTx3042 and PI291382 were separated from others and showed increased PS II quantum yield (0.74 to 0.77), SPAD (50 to 60) and HI (0.30 to 0.47) with early maturity (61 to 66 days to flowering) under dryland condition (Additional file 2: Table S3). Likewise, genotype IS24463 showed increased tolerance to both pathogens. Combined PCA analysis of drought and disease related traits clearly indicated that chlorophyll content, PS II quantum yield and HI can be used as a screening index for post-flowering drought and disease tolerance (Figure 3). The aforementioned sources can be used to develop potential lines for post-flowering drought tolerance, Fusarium stalk and charcoal rot resistance.

## Conclusions

Wide variability was observed between the genotypes for chlorophyll content, PS II quantum yield and leaf temperature as well as HI and Fusarium stalk rot and charcoal rot lesion length and grain yield in the sorghum minicore germplasm. Genotypes PI510898, IS1212 and PI533946 showed increased grain yield; modest values of chlorophyll content, PS II quantum yield, and leaf temperature; and early maturity under dryland conditions. Genotypes IS14290, IS12945 and IS1219 showed greater susceptibility to drought with lower grain yield and HI along with late maturity. Genotype IS24463 expressed a high degree of tolerance to both Fusarium stalk rot and charcoal rot and IS12706 and IS16151 were highly susceptible based on mean lesion length and grain yield under dryland conditions. Lesion length and grain yield were negatively correlated with chlorophyll content, PS II quantum yield and HI under both diseases indicating that higher pathogen infection resulted in lower plant health and reduced grain yield. Genotype PI510898 showed combined tolerance to drought and Fusarium stalk rot under dryland conditions. The aforementioned genotypes can be a potential sources for improvement of drought and disease tolerance in future sorghum breeding.

## Electronic supplementary material

Additional file 1: Figure S1: Precipitation and average temperature for Hays, KS, during the crop growth period from June to October 2011. *Note:* *National Weather Service 30-year average (1981 to 2010); “1^st^ (40 DAP)”, “2^nd^ (52 DAP)”, and “3^rd^ (86 DAP)”. (DOCX 64 KB)

Additional file 2: Table S1: Mean square and significance levels for agronomic and drought related traits in sorghum exotic germplasm and adapted lines. **Table S2.** Mean squares and significance levels (*p*) from ANOVA for lesion length and grain yield related to Fusarium stalk rot and charcoal rot in sorghum exotic germplasm and adapted lines. **Table S3.** Mean performance of genotypes for physiological traits and grain yield under dryland and irrigated environments. (JPEG 37 KB)

## Abbreviations

PCA: Principal component analysis
DAP: Days after planting
VPD: Vapor pressure deficit.
