# Supplementary material for: Genotypic variation in sorghum [Sorghum bicolor (L.) Moench] exotic germplasm collections for drought and disease tolerance
Source: Springerplus. 2013 Dec 4;2:650. doi: 10.1186/2193-1801-2-650 (PMC3863401; doi:10.1186/2193-1801-2-650)
Supplement: Supplementary file 1 — Additional file 1: Figure S1: Precipitation and average temperature for Hays, KS, during the crop growth period from June to October 2011. Note: *National Weather Service 30-year average (1981 to 2010); “1st (40 DAP)”, “2nd (52 DAP)”, and “3rd (86 DAP)”. (DOCX 64 KB) [file 40064_2013_704_MOESM1_ESM.docx]

**Supplementary table S1 Mean square and significance levels for agronomic and drought related traits in sorghum exotic germplasm and adapted lines**

| Source | df | SPAD | | |  | PS II quantum yield | |  | LT | |  |  | Plant  height | Days to flowering |
| --- | --- | --- | --- | --- | --- | --- | --- | --- | --- | --- | --- | --- | --- | --- |
|  |  | (DAP) | | |  | (DAP) | |  | (DAP) | |  |  |  |  |
|  |  | 59 | 76 | 103 |  | 67 | 83 |  | 61 | 81 |  | df |  |  |
| **Dryland** |  |  |  |  |  |  |  |  |  |  |  |  |  |  |
| Block | 1 | 103.52 | 1065.38*** | 3656.95*** |  | 0.05*** | 0.50*** |  | 2908.48*** | 210.04*** |  | 1 | 2406.61 | 20.42 |
| Entry | 138 | 165.66*** | 247.76*** | 351.10*** |  | 0.01*** | 0.04*** |  | 12.08*** | 12.97 |  | 139 | 4667.88*** | 266.30*** |
| Error | 686 | 34.05 | 56.70 | 74.76 |  | 0.001 | 0.001 |  | 4.58 | 13.79 |  | 137 | 979.51 | 33.79 |
| RE |  | 0.79 | 0.77 | 0.79 |  | 0.88 | 0.95 |  | 0.62 | - |  |  | 0.79 | 0.87 |
| **Irrigated** |  |  |  |  |  |  |  |  |  |  |  |  |  |  |
| Block | 1 | 117.19 | 68.84 | 235.38* |  | 0.02** | 0.03*** |  | 2256.39*** | 3523.67*** |  | 1 | 1.47 | 130.62* |
| Entry | 138 | 212.49*** | 284.89*** | 84.61*** |  | 0.01*** | 0.01* |  | 156.18 | 10.15*** |  | 139 | 10340.52*** | 167.76*** |
| Error | 672 (409†) | 39.35 | 55.22 | 35.78 |  | 0.001 | 0.001 |  | 152.10 | 3.82 |  | 136  ( 131^¶^) | 1367.64 | 23.17 |
| RE |  | 0.81 | 0.81 | 0.99 |  | 0.99 | 0.99 |  | 0.03 | 0.62 |  |  | 0.87 | 0.86 |

†Error df for SPAD at 103 DAP; ^¶^Error df for days to flowering; * *P* < 0.05, ** *P* < 0.01, and *** *P* < 0.001 significance level; -negative value

DAP = days after planting; SPAD = chlorophyll content; PS II quantum yield = chlorophyll fluorescence; LT = Leaf temperature; RE = Repeatability estimate

**Supplementary table S2** **Mean squares and significance levels (*p*) from ANOVA for lesion length and grain yield related to Fusarium stalk rot and charcoal rot in sorghum exotic germplasm and adapted lines**

| Source | Lesion length | | |  | Grain yield | | | |
| --- | --- | --- | --- | --- | --- | --- | --- | --- |
|  | df | Stalk rot | Charcoal rot |  | df | Stalk rot | Charcoal rot | Control |
| **Dryland** |  |  |  |  |  |  |  |  |
| Block | 1 | 90.1 | 12.28 |  | 1 | 938.02** | 372.16 | 32.04 |
| Entry | 93 | 163.56*** | 187.53*** |  | 96 | 634.88*** | 515.91*** | 251.81** |
| Error | 357 | 31.14 | 45.07 |  | 57 | 117.59 | 211.57 | 125.79 |
| RE |  | 0.81 | 0.75 |  |  | 0.81 | 0.58 | 0.50 |
|  |  |  |  |  |  |  |  |  |
| **Irrigated** |  |  |  |  |  |  |  |  |
| Block | 1 | 206.3 | 9.71 |  | 1 | 23.85 | 14.54 | 31.29 |
| Entry | 125 | 209.55*** | 171.62*** |  | 125 | 494.21*** | 694.68*** | 485.01*** |
| Error | 309 | 56.51 | 56.24 |  | 84 | 259.29 | 340.57 | 229.08 |
| RE |  | 0.73 | 0.67 |  |  | 0.47 | 0.51 | 0.53 |

** *P* < 0.01 and *** *P* < 0.001 significance level; *Note:* Stalk rot *(F. thapsinum);* Charcoal rot *(M. phaseolina).*

RE = Repeatability estimate

**Supplementary table S3** **Mean performance of genotypes for physiological traits and grain yield under dryland and irrigated environment.**

|  |  | Dryland | | | | | | |  | Irrigated | | | | | |  |
| --- | --- | --- | --- | --- | --- | --- | --- | --- | --- | --- | --- | --- | --- | --- | --- | --- |
| Entry | Pedigree | Chloro- phyll  content  (SPAD) | PS II quant-um yield | LT (°C) | PHT  (cm) | FLD  (day) | HI | Grain yield  (g  plant^-1^) |  | Chloro-phyll content (SPAD) | PS II quant-um yield | LT  (°C) | PHT  (cm) | FLD  (day) | HI | Grain yield  (g plant^-1^) |
| 1 | IS608 | 38.46 | 0.74 | 36.56 | 200 | 95 | 0.06 | 4.73 |  | 38.68 | 0.73 | 36.79 | 260 | 80 | 0.06 | 14.50 |
| 2 | IS995 | 34.50 | 0.75 | 38.61 | 210 | 91 | 0.07 | 13.23 |  | 43.08 | 0.73 | 34.29 | 268 | 88 | 0.07 | 24.77 |
| 3 | IS1212 | 46.31 | 0.74 | 38.73 | 235 | 77 | 0.45 | 58.10 |  | 56.31 | 0.78 | 36.42 | 275 | 75 | - | - |
| 4 | IS1219 | 34.08 | 0.71 | 39.22 | 250 | 80 | 0.00 | 0.20 |  | 63.65 | 0.73 | 35.79 | 290 |  | - | - |
| 5 | IS1233 | 39.19 | 0.76 | 38.38 | 205 | 66 | 0.14 | 8.77 |  | 49.65 | 0.74 | 37.18 | 248 | 72 | - | - |
| 6 | IS2205 | 38.56 | 0.74 | 39.06 | 220 | 98 | 0.20 | 24.30 |  | 44.40 | 0.76 | 38.64 | 268 | 92 | 0.16 | 56.53 |
| 7 | IS2389 | 52.29 | 0.74 | 39.13 | 208 | 79 | 0.38 | 42.30 |  | 53.10 | 0.72 | 37.75 | 250 | 79 | 0.11 | 49.50 |
| 8 | IS2397 | 44.92 | 0.73 | 41.40 | 140 | 90 | 0.18 | 15.53 |  | 44.13 | 0.75 | 36.65 | 188 | 85 | 0.30 | 42.27 |
| 9 | IS2426 | 41.31 | 0.67 | 38.21 | 195 | 86 | 0.08 | 6.80 |  | 44.32 | 0.73 | 37.12 | 290 | 83 | - | 13.27 |
| 10 | IS2864 | 46.13 | 0.74 | 37.92 | 175 | 88 | 0.02 | 3.17 |  | 45.37 | 0.75 | 38.09 | 253 | 91 | 0.18 | 49.90 |
| 11 | IS2872 | 51.03 | 0.75 | 38.98 | 130 | 70 | 0.47 | 51.63 |  | 57.73 | 0.75 | 35.83 | 158 | 72 | 0.28 | 54.77 |
| 12 | IS3946 | 43.14 | 0.73 | 38.83 | 225 | 94 | 0.04 | 3.80 |  | 52.00 | 0.75 | 35.77 | 345 | 90 | 0.08 | 15.90 |
| 13 | IS3971 | 42.06 | 0.76 | 38.16 | 213 | 79 | 0.52 | 32.13 |  | 50.00 | 0.75 | 35.48 | 273 | 87 | 0.16 | 19.87 |
| 14 | IS4515 | 37.57 | 0.74 | 37.61 | 180 | 91 | 0.07 | 14.00 |  | 49.34 | 0.77 | 36.98 | 300 | 89 | 0.15 | 67.53 |
| 15 | IS4631 | 42.21 | 0.76 | 38.19 | 238 | 81 | 0.38 | 30.10 |  | 53.10 | 0.75 | 36.93 | 270 | 85 | 0.12 | 54.30 |
| 16 | IS4698 | 42.35 | 0.73 | 38.10 | 146 | 90 | 0.20 | 21.30 |  | 52.23 | 0.73 | 36.02 | 295 | 88 | 0.15 | 49.20 |
| 17 | IS5094 | 40.72 | 0.75 | 36.83 | 238 | 96 | 0.14 | 14.37 |  | 54.70 | 0.77 | 36.53 | 315 | 89 | 0.13 | 27.10 |
| 18 | IS8348 | 43.04 | 0.75 | 40.31 | 223 | 78 | 0.40 | 27.83 |  | 47.53 | 0.75 | 34.86 | 230 | 75 | 0.27 | 61.07 |
| 19 | IS8777 | 45.02 | 0.75 | 38.05 | 178 | 108 | 0.14 | 11.90 |  | 48.50 | 0.73 | 36.91 | 230 | 104 | 0.15 | 9.13 |
| 20 | IS12302 | 42.82 | 0.74 | 39.34 | 220 | - | 0.01 | 1.97 |  | 48.73 | 0.75 | 38.64 | 298 | 98 | 0.11 | 36.03 |
| 21 | IS12706 | 43.40 | 0.76 | 39.94 | 133 | 59 | 0.37 | 20.90 |  | 47.71 | 0.77 | 36.32 | 230 | 65 | 0.43 | 27.50 |
| 22 | IS12735 | 42.11 | 0.73 | 37.29 | 190 | 74 | 0.37 | 23.80 |  | 47.36 | 0.73 | 34.79 | 245 | 75 | 0.29 | 57.47 |
| 23 | IS12804 | 38.93 | 0.74 | 40.77 | 185 | 63 | 0.28 | 13.07 |  | 47.38 | 0.76 | 35.11 | 238 | 60 | 0.22 | 20.43 |
| 24 | IS12883 | 39.22 | 0.74 | 40.02 | 248 | 92 | 0.22 | 18.77 |  | 50.75 | 0.70 | 34.96 | 313 | 81 | 0.36 | 67.60 |
| 25 | IS12945 | 38.93 | 0.76 | 39.58 | 195 | 96 | 0.00 | 0.17 |  | 51.77 | 0.76 | 36.48 | 303 | 92 | 0.12 | 46.90 |
| 26 | IS13782 | 50.57 | 0.72 | 38.11 | 133 | 82 | 0.17 | 22.73 |  | 49.96 | 0.73 | 36.49 | 213 | 79 | 0.35 | 47.50 |
| 27 | IS14010 | 47.92 | 0.73 | 40.60 | 125 | 70 | 0.46 | 27.03 |  | 49.26 | 0.76 | 34.91 | 168 | 77 | 0.16 | 26.30 |
| 28 | IS14090 | 50.63 | 0.71 | 38.27 | 120 | 80 | 0.26 | 12.80 |  | 49.18 | 0.76 | 36.93 | 135 | 80 | 0.32 | 43.37 |
| 29 | IS14290 | 40.66 | 0.76 | 38.39 | 185 | 105 | 0.00 | 0.07 |  | 47.10 | 0.75 | 35.98 | 328 | 96 | 0.14 | 40.60 |
| 30 | IS17941 | 39.77 | 0.72 | 39.05 | 163 | 95 | 0.23 | 23.37 |  | 43.21 | 0.74 | 36.87 | 190 | 95 | 0.20 | 46.97 |
| 31 | IS19389 | 39.03 | 0.72 | 40.48 | 183 | 102 | 0.04 | 7.17 |  | 42.60 | 0.75 | 34.77 | 263 | 89 | 0.08 | 18.07 |
| 32 | IS19445 | 50.86 | 0.76 | 39.73 | 138 | 83 | 0.31 | 33.37 |  | 51.48 | 0.76 | 36.76 | 165 | 77 | 0.11 | 32.77 |
| 33 | IS19450 | 46.76 | 0.73 | 38.20 | 210 | 84 | 0.34 | 34.73 |  | 43.91 | 0.75 | 36.79 | 243 | 81 | 0.19 | 56.97 |
| 34 | IS20697 | 41.91 | 0.73 | 39.50 | 168 | 82 | 0.47 | 36.17 |  | 42.51 | 0.75 | 35.14 | 183 | 84 | 0.25 | 42.40 |
| 35 | IS20816 | 37.73 | 0.74 | 37.73 | 185 | 97 | 0.05 | 2.80 |  | 46.85 | 0.76 | 37.13 | 250 | 85 | - | 7.23 |
| 36 | IS21863 | 54.32 | 0.73 | 38.42 | 140 | 62 | 0.43 | 20.37 |  | 56.48 | 0.76 | 35.63 | 195 | 63 | 0.28 | 33.70 |
| 37 | IS22294 | 44.68 | 0.75 | 38.33 | 228 | 105 | 0.13 | 20.53 |  | 49.63 | 0.74 | 35.43 | 303 | 93 | 0.15 | 26.07 |
| 38 | IS22616 | 43.58 | 0.76 | 38.54 | 213 | 101 | 0.03 | 2.00 |  | 44.76 | 0.77 | 36.48 | 318 | 90 | 0.17 | 36.23 |
| 39 | IS23992 | 44.49 | 0.76 | 38.72 | 298 | 105 | - | 6.57 |  | 51.30 | 0.78 | 35.72 | 365 | 91 | 0.09 | 32.30 |
| 40 | IS24348 | 37.18 | 0.73 | 39.88 | 150 | 100 | 0.13 | 24.37 |  | 45.74 | 0.75 | 37.90 | 190 | 84 | 0.14 | 36.57 |
| 41 | IS24365 | 44.94 | 0.76 | 39.88 | 190 | 85 | 0.19 | 28.83 |  | 50.36 | 0.75 | 36.77 | 328 | 99 | 0.09 | 54.87 |
| 42 | IS24453 | 44.18 | 0.74 | 41.56 | 218 | 88 | 0.19 | 15.00 |  | 45.80 | 0.73 | 36.88 | 265 | 86 | 0.13 | 49.23 |
| 43 | IS24463 | 40.96 | 0.74 | 38.02 | 243 | 90 | 0.30 | 33.97 |  | 48.34 | 0.74 | 36.65 | 310 | 88 | 0.21 | 30.73 |
| 44 | IS24492 | 40.14 | 0.73 | 38.66 | 178 | 95 | 0.01 | 1.97 |  | 43.19 | 0.71 | 37.17 | 298 | 93 | 0.13 | 55.03 |
| 45 | IS26694 | 43.66 | 0.74 | 36.46 | 175 | 108 | 0.00 | 12.00 |  | 47.54 | 0.74 | 36.50 | 280 | 97 | 0.13 | 45.07 |
| 46 | IS26701 | 36.37 | 0.74 | 40.32 | 180 | 94 | 0.06 | 9.43 |  | 43.20 | 0.69 | 38.56 | 220 | 83 | 0.11 | 39.03 |
| 47 | IS26737 | 41.41 | 0.77 | 38.64 | 245 | 91 | 0.26 | 37.73 |  | 51.51 | 0.74 | 36.40 | 305 | 87 | 0.19 | 70.23 |
| 48 | IS26749 | 47.19 | 0.75 | 37.45 | 140 | 89 | 0.35 | 35.33 |  | 49.41 | 0.76 | 36.29 | 145 | 82 | 0.29 | 35.63 |
| 49 | IS28449 | 44.28 | 0.77 | 38.48 | 275 | 83 | 0.17 | 13.60 |  | 45.04 | 0.75 | 36.35 | 348 | 76 | 0.11 | 15.27 |
| 50 | IS28451 | 42.33 | 0.79 | 37.47 | 238 | 75 | 0.09 | 4.83 |  | 51.46 | 0.77 | 35.12 | 280 | 73 | 0.04 | 9.02 |
| 51 | IS28614 | 44.03 | 0.77 | 36.58 | 270 | 72 | 0.03 | 1.47 |  | 51.56 | 0.76 | 37.35 | 290 | 66 | 0.15 | 32.63 |
| 52 | IS29187 | 38.78 | 0.74 | 40.74 | 188 | - | 0.04 | 6.83 |  | 46.47 | 0.73 | 35.74 | 303 | 99 | 0.04 | 5.78 |
| 53 | IS29233 | 46.37 | 0.76 | 39.35 | 115 | 64 | 0.13 | 23.60 |  | 45.39 | 0.68 | 37.29 | 150 | 100 | 0.11 | 27.63 |
| 54 | IS29304 | 37.02 | 0.71 | 39.73 | 165 | - | 0.07 | 15.43 |  | 45.55 | 0.72 | 38.36 | 303 | 99 | 0.13 | 52.23 |
| 55 | IS29314 | 38.91 | 0.73 | 38.49 | 210 | 110 | - | - |  | 47.63 | 0.75 | 36.75 | 330 | 82 | 0.14 | 39.57 |
| 56 | IS29326 | 50.33 | 0.74 | 40.84 | 160 | 101 | 0.25 | 37.83 |  | 25.04 | 0.61 | 42.46 | 208 | 95 | 0.09 | 27.47 |
| 57 | IS29335 | 42.62 | 0.74 | 38.30 | 203 | 93 | 0.16 | 28.87 |  | 53.78 | 0.73 | 37.53 | 253 | 89 | 0.11 | 56.27 |
| 58 | IS29358 | 38.39 | 0.73 | 40.28 | 193 | 99 | 0.10 | 11.47 |  | 49.35 | 0.72 | 35.62 | 280 | 90 | 0.21 | 53.73 |
| 59 | IS29468 | 43.62 | 0.71 | 36.83 | 188 | 96 | 0.05 | 4.57 |  | 43.93 | 0.69 | 36.33 | 288 | 93 | 0.16 | 64.60 |
| 60 | IS59519 | 41.52 | 0.72 | 40.18 | 205 | 94 | 0.08 | 7.83 |  | 47.03 | 0.72 | 36.03 | 240 | 90 | 0.16 | 37.90 |
| 61 | IS29582 | 43.83 | 0.74 | 39.88 | 118 | 91 | 0.06 | 8.97 |  | 53.38 | 0.74 | 37.17 | 155 | 85 | 0.22 | 37.57 |
| 62 | IS29627 | 43.99 | 0.73 | 39.37 | 218 | 86 | 0.24 | 12.17 |  | 53.78 | 0.77 | 35.95 | 283 | 83 | 0.24 | 46.47 |
| 63 | IS29654 | 33.68 | 0.74 | 36.44 | 250 | 87 | 0.16 | 7.77 |  | 45.35 | 0.75 | 37.22 | 290 | 79 | 0.04 | 1.16 |
| 64 | IS29689 | 44.17 | 0.76 | 36.67 | 203 | 104 | 0.02 | 17.57 |  | 51.82 | 0.74 | 36.13 | 240 | 95 | 0.15 | 50.57 |
| 65 | IS29733 | 41.89 | 0.74 | 40.88 | 183 | 102 | 0.04 | 4.04 |  | 50.32 | 0.75 | 36.52 | 245 | 89 | 0.18 | 39.57 |
| 66 | IS30231 | 40.96 | 0.75 | 39.36 | 180 | - | 0.06 | 10.00 |  | 49.11 | 0.73 | 35.78 | 328 | 95 | - | 43.70 |
| 67 | IS30383 | 43.11 | 0.74 | 39.66 | 233 | 79 | 0.23 | 22.13 |  | 52.34 | 0.75 | 36.83 | 245 | 72 | 0.19 | 37.83 |
| 68 | IS30507 | 43.31 | 0.77 | 39.63 | 210 | 78 | 0.32 | 17.63 |  | 48.04 | 0.73 | 36.32 | 265 | 78 | 0.16 | 25.50 |
| 69 | IS30508 | 54.07 | 0.77 | 38.65 | 183 | 74 | 0.32 | 15.60 |  | 53.59 | 0.73 | 36.12 | 218 | 80 | 0.37 | 55.80 |
| 70 | IS30533 | 43.49 | 0.74 | 39.73 | 240 | 78 | 0.29 | 16.57 |  | 44.94 | 0.73 | 38.04 | 280 | 81 | 0.19 | 35.77 |
| 71 | IS30536 | 38.65 | 0.74 | 40.53 | 183 | 79 | 0.31 | 15.47 |  | 47.18 | 0.76 | 36.33 | 278 | 77 | 0.14 | 22.53 |
| 72 | IS30562 | 44.39 | 0.72 | 39.60 | 183 | 87 | 0.48 | 43.60 |  | 49.86 | 0.76 | 35.46 | 220 | 84 | 0.19 | 50.70 |
| 73 | IS32295 | 39.58 | 0.73 | 38.40 | 230 | 102 | 0.06 | 6.27 |  | 47.11 | 0.76 | 34.30 | 325 | 95 | 0.12 | 34.50 |
| 74 | IS33844 | 38.21 | 0.75 | 38.80 | 208 | 99 | 0.04 | 3.60 |  | 53.35 | 0.77 | 35.54 | 325 | 91 | 0.09 | 66.60 |
| 75 | IS473 | 39.21 | 0.75 | 38.91 | 190 | 102 | 0.03 | 2.90 |  | 46.31 | 0.76 | 35.72 | 265 | 92 | 0.17 | 29.60 |
| 76 | IS602 | 42.39 | 0.77 | 38.42 | 235 | 79 | 0.18 | 17.30 |  | 47.60 | 0.73 | 37.19 | 210 | 80 | 0.15 | 30.13 |
| 77 | IS27912 | 48.99 | 0.75 | 38.73 | 148 | 93 | 0.08 | 10.77 |  | 51.82 | 0.74 | 35.89 | 198 | 86 | 0.07 | 12.40 |
| 78 | IS20743 | 44.83 | 0.75 | 40.18 | 238 | 76 | 0.13 | 6.03 |  | 51.89 | 0.77 | 34.60 | 308 | 65 | 0.11 | 16.00 |
| 79 | IS20727 | 51.51 | 0.75 | 38.28 | 173 | 61 | 0.34 | 34.53 |  | 54.47 | 0.76 | 35.22 | 230 | 73 | 0.25 | 58.34 |
| 80 | IS19676 | 48.69 | 0.74 | 40.13 | 143 | 99 | . | 4.63 |  | 52.78 | 0.73 | 35.93 | 198 | 96 | 0.35 | 38.50 |
| 81 | IS16151 | 37.69 | 0.73 | 37.92 | 238 | 84 | 0.20 | 17.30 |  | 45.99 | 0.74 | 35.81 | 290 | 75 | 0.11 | 18.57 |
| 82 | IS19262 | 52.67 | 0.77 | 38.58 | 161 | 61 | 0.42 | 18.60 |  | 52.77 | 0.76 | 36.43 | 73 | 68 | 0.30 | 20.97 |
| 83 | ICSR 89058 | 48.63 | 0.77 | 38.53 | 155 | 72 | 0.45 | 31.20 |  | 47.52 | 0.75 | 35.12 | 135 | 82 | 0.39 | 53.47 |
| 84 | IS4581 | 42.61 | 0.75 | 39.53 | 213 | 95 | 0.10 | 20.80 |  | 48.12 | 0.75 | 36.44 | 258 | 90 | 0.14 | 54.80 |
| 85 | PI257309 | 47.64 | 0.76 | 36.55 | 250 | 80 | 0.29 | 22.57 |  | 52.94 | 0.75 | 36.53 | 268 | 80 | 0.18 | 33.73 |
| 86 | PI295121 | 47.01 | 0.73 | 40.73 | 155 | 78 | 0.14 | 9.87 |  | 47.48 | 0.72 | 35.64 | 138 | 78 | 0.04 | 2.45 |
| 87 | PI236278 | 53.72 | 0.76 | 37.89 | 128 | 69 | 0.47 | 22.37 |  | 49.38 | 0.76 | 36.80 | 228 | 71 | 0.30 | 29.60 |
| 88 | PI510898 | 55.14 | 0.76 | 38.07 | 140 | 85 | 0.36 | 66.40 |  | 50.34 | 0.76 | 38.26 | 163 | 80 | 0.36 | 41.47 |
| 89 | PI510920 | 46.93 | 0.75 | 38.30 | 158 | 85 | 0.47 | 43.00 |  | 46.91 | 0.73 | 34.56 | 178 | 83 | 0.23 | 32.37 |
| 90 | PI291382 | 49.51 | 0.74 | 40.77 | 208 | 60 | 0.30 | 12.67 |  | 49.71 | 0.76 | 35.20 | 235 | 67 | 0.32 | 56.87 |
| 91 | PI408822 | 44.12 | 0.74 | 38.37 | 260 | 83 | 0.26 | 25.03 |  | 41.20 | 0.72 | 35.81 | 318 | 83 | 0.31 | 77.97 |
| 92 | PI548007 | 43.16 | 0.77 | 39.17 | 210 | 83 | 0.29 | 17.73 |  | 51.19 | 0.76 | 37.08 | 240 | 80 | 0.15 | 45.20 |
| 93 | PI548034 | 43.12 | 0.76 | 37.06 | 205 | 70 | 0.25 | 23.87 |  | 49.53 | 0.75 | 37.43 | 223 | 75 | 0.32 | 50.60 |
| 94 | PI391652 | 42.18 | 0.73 | 38.78 | 186 | 71 | 0.39 | 29.23 |  | 49.11 | 0.71 | 37.32 | 188 | 70 | 0.37 | 35.93 |
| 95 | PI610730 | 40.87 | 0.75 | 38.19 | 258 | 77 | 0.16 | 18.70 |  | 47.08 | 0.78 | 36.99 | 268 | 72 | 0.17 | 38.50 |
| 96 | PI276797 | 43.22 | 0.74 | 37.26 | 208 | 70 | 0.15 | 7.57 |  | 50.34 | 0.77 | 36.60 | 263 | 74 | 0.21 | 30.27 |
| 97 | PI576380 | 48.33 | 0.76 | 37.57 | 155 | 79 | 0.30 | 25.27 |  | 50.32 | 0.77 | 37.26 | 138 | 84 | 0.26 | 40.30 |
| 98 | PI262568 | 48.55 | 0.74 | 38.99 | 168 | 67 | 0.33 | 38.93 |  | 52.78 | 0.75 | 36.91 | 258 | 68 | 0.31 | 47.47 |
| 99 | PI267109 | 60.27 | 0.77 | 40.58 | 118 | 60 | 0.40 | 36.67 |  | 59.31 | 0.75 | 37.37 | 155 | 64 | 0.44 | 57.27 |
| 100 | PI550685 | 51.81 | 0.75 | 36.90 | 123 | 78 | 0.36 | 20.53 |  | 51.56 | 0.73 | 38.49 | 163 | 70 | 0.32 | 45.70 |
| 101 | PI585330 | 45.08 | 0.77 | 38.68 | 158 | 61 | 0.11 | 10.40 |  | 55.23 | 0.77 | 35.34 | 200 | 66 | 0.18 | 21.30 |
| 102 | PI586440 | 37.63 | 0.74 | 36.83 | 135 | 81 | 0.21 | 17.73 |  | 40.99 | 0.74 | 37.27 | 145 | 87 | 0.30 | 52.23 |
| 103 | PI267392 | 53.82 | 0.73 | 37.47 | 175 | 83 | 0.30 | 34.90 |  | 49.83 | 0.75 | 36.89 | 188 | 80 | 0.23 | 44.93 |
| 104 | PI267379 | 45.56 | 0.77 | 37.22 | 90 | 77 | 0.25 | 18.57 |  | 50.44 | 0.76 | 36.24 | 80 | 77 | 0.25 | 42.00 |
| 105 | PI533946 | 53.66 | 0.77 | 39.75 | 80 | 76 | 0.42 | 58.03 |  | 48.99 | 0.77 | 37.91 | 85 | 76 | - | 11.23 |
| 106 | PI562891 | 40.49 | 0.76 | 38.28 | 203 | 87 | 0.23 | 13.67 |  | 48.38 | 0.75 | 35.61 | 295 | 90 | 0.15 | 27.53 |
| 107 | PI536516 | 47.35 | 0.78 | 38.03 | 170 | 90 | 0.22 | 22.30 |  | 45.95 | 0.75 | 37.29 | 225 | 90 | 0.17 | 36.30 |
| 108 | PI264451 | 61.92 | 0.75 | 37.44 | 128 | 62 | 0.19 | 38.20 |  | 58.45 | 0.75 | 38.14 | 173 | 69 | 0.45 | 47.10 |
| 109 | PI534138 | 45.40 | 0.74 | 37.79 | 95 | 84 | 0.01 | 10.00 |  | 48.70 | 0.77 | 37.46 | 125 | 73 | 0.33 | 31.43 |
| 110 | PI562166 | 44.49 | 0.71 | 38.38 | 155 | 75 | 0.43 | 50.30 |  | 50.64 | 0.75 | 35.01 | 163 | 74 | 0.33 | 53.57 |
| 111 | PI550590 | 51.17 | 0.74 | 38.68 | 108 | 64 | 0.39 | 51.87 |  | 52.21 | 0.77 | 34.23 | 125 | 69 | 0.40 | 42.50 |
| 112 | PI534052 | 45.26 | 0.76 | 38.53 | 105 | 81 | 0.35 | 24.37 |  | 39.54 | 0.77 | 35.69 | 113 | 83 | 0.32 | 49.17 |
| 113 | PI591002 | 47.11 | 0.75 | 38.59 | 110 | 81 | 0.18 | 16.03 |  | 46.49 | 0.76 | 35.51 | 123 | 80 | 0.25 | 30.47 |
| 114 | PI562723 | 39.61 | 0.76 | 42.03 | 168 | 80 | 0.34 | 22.33 |  | 47.26 | 0.73 | 35.26 | 255 | 70 | 0.04 | 14.67 |
| 115 | PI475432 | 47.70 | 0.77 | 41.49 | 160 | 83 | 0.57 | 34.43 |  | 46.82 | 0.74 | 35.13 | 213 | 82 | 0.16 | 39.03 |
| 116 | PI533916 | 49.92 | 0.74 | 39.53 | 98 | 61 | 0.47 | 20.77 |  | 49.34 | 0.76 | 35.87 | 93 | 81 | - | 9.47 |
| 117 | PI565174 | 42.66 | 0.76 | 39.73 | 123 | 92 | 0.09 | 9.70 |  | 45.51 | 0.76 | 35.07 | 153 | 90 | 0.22 | 28.77 |
| 118 | BTx399 | 56.34 | 0.76 | 39.75 | 73 | 77 | 0.32 | 32.43 |  | 43.92 | 0.72 | 36.75 | 78 | 77 | 0.23 | 29.93 |
| 119 | Laing TangAi | 43.02 | 0.75 | 39.56 | 112 | 73 | 0.46 | 34.83 |  | 47.03 | 0.69 | 38.11 | 108 | 81 | 0.10 | 9.27 |
| 120 | Ajabsido | 46.36 | 0.73 | 34.83 | 145 | 73 | 0.37 | 27.20 |  | 52.71 | 0.74 | 36.18 | 158 | 75 | 0.29 | 71.13 |
| 121 | PI570959 | 51.08 | 0.76 | 39.04 | 178 | 71 | 0.43 | 22.40 |  | 51.84 | 0.76 | 35.74 | 235 | 72 | 0.20 | 53.01 |
| 122 | PI570895 | 52.30 | 0.75 | 36.38 | 175 | 73 | 0.40 | 43.30 |  | 49.96 | 0.74 | 36.04 | 200 | 72 | 0.26 | 55.30 |
| 123 | PI568992 | 39.98 | 0.75 | 40.98 | 150 | 87 | 0.02 | 1.33 |  | 41.98 | 0.76 | 36.46 | 248 | 85 | 0.34 | 61.53 |
| 124 | PI571032 | 40.04 | 0.72 | 38.19 | 183 | 77 | 0.25 | 13.73 |  | 50.05 | 0.77 | 37.37 | 250 | 74 | 0.20 | 24.60 |
| 125 | PI563146 | 44.33 | 0.74 | 39.90 | 180 | 82 | 0.05 | 3.90 |  | 47.61 | 0.75 | 36.88 | 220 | 78 | 0.12 | 31.07 |
| 126 | PI571165 | 47.86 | 0.72 | 38.33 | 170 | 77 | 0.37 | 40.73 |  | 50.40 | 0.75 | 36.03 | 218 | 77 | 0.28 | 45.20 |
| 127 | PI569810 | 38.76 | 0.74 | 39.46 | 156 | 79 | 0.01 | 0.63 |  | 39.57 | 0.76 | 37.67 | 198 | 77 | 0.22 | 39.20 |
| 128 | PI568323 | 37.81 | 0.74 | 41.08 | 210 | 83 | 0.44 | 18.07 |  | 44.39 | 0.74 | 37.64 | 320 | 87 | 0.14 | 38.83 |
| 129 | PI569809 | 52.35 | 0.77 | 38.08 | 153 | 79 | 0.30 | 47.43 |  | 54.48 | 0.76 | 36.08 | 268 | 86 | 0.23 | 62.03 |
| 130 | PI534052 | 42.90 | 0.76 | 39.64 | 118 | 78 | 0.28 | 17.87 |  | 35.38 | 0.73 | 36.60 | 93 | 87 | 0.19 | 8.23 |
| 131 | PI563253 | 40.47 | 0.71 | 42.38 | 103 | 88 | - | - |  | 48.03 | 0.75 | 35.84 | 163 | 83 | 0.26 | 57.40 |
| 132 | KS19R | 50.39 | 0.76 | 35.88 | 100 | 78 | 0.44 | 24.33 |  | 52.93 | 0.76 | 36.61 | 143 | 67 | 0.31 | 55.70 |
| 133 | SC599R | 45.40 | 0.76 | 38.24 | 153 | 64 | - | 17.00 |  | 58.21 | 0.75 | 39.10 | 100 | 66 | - | - |
| 134 | 1790E | 50.97 | 0.77 | 38.64 | 102 | 69 | 0.32 | 31.37 |  | 52.88 | 0.78 | 39.50 | 85 | 69 | 0.05 | 2.75 |
| 135 | BTx642 | 48.97 | 0.78 | 39.53 | 73 | 83 | 0.46 | 42.33 |  | 49.90 | 0.75 | 37.17 | 145 | 91 | - | - |
| 136 | SC35R | 47.32 | 0.75 | 39.90 | 123 | 94 | 0.13 | 15.57 |  | 48.55 | 0.75 | 35.28 | 95 | 92 | 0.03 | 7.30 |
| 137 | SC399B | 22.67 | 0.68 | 40.62 | 60 | 103 | - | - |  | 50.29 | 0.79 | 38.32 | 95 | 82 | - | - |
| 138 | BTx3042 | 56.75 | 0.74 | 39.20 | 80 | 61 | 0.34 | 46.87 |  | 57.23 | 0.77 | 35.98 | 85 | 67 | 0.04 | 3.87 |
| 139 | RTx7078 | 51.46 | 0.75 | 39.48 | 103 | 70 | 0.42 | 30.63 |  | 48.53 | 0.77 | 38.33 | 103 | 73 | 0.29 | 22.60 |
| 140 | RTx7000 | 49.07 | 0.78 | 42.38 | 83 | 76 | 0.33 | 30.40 |  | 51.96 | 0.78 | 35.48 | 100 | 79 | 0.36 | 40.10 |

Chlorophyll content measured by SPAD meter; LT (°C) = leaf temperature; PHT = plant height (cm); FLD= flowering days; HI = harvest index.
