# Supplementary figures and images for: Genotypic variation in sorghum [Sorghum bicolor (L.) Moench] exotic germplasm collections for drought and disease tolerance
Source: Springerplus. 2013 Dec 4;2:650. doi: 10.1186/2193-1801-2-650 (PMC3863401; doi:10.1186/2193-1801-2-650)

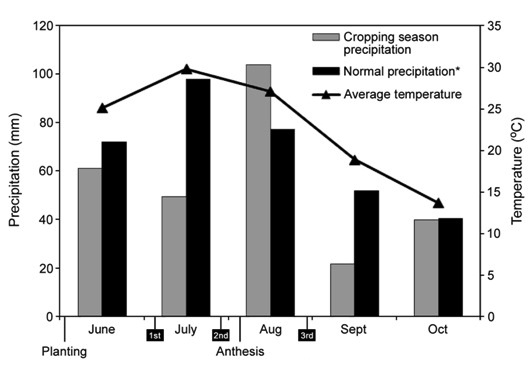

Supplement: Supplementary file 2 — Additional file 2: Table S1: Mean square and significance levels for agronomic and drought related traits in sorghum exotic germplasm and adapted lines. Table S2. Mean squares and significance levels (p) from ANOVA for lesion length and grain yield related to Fusarium stalk rot and charcoal rot in sorghum exotic germplasm and adapted lines. Table S3. Mean performance of genotypes for physiological traits and grain yield under dryland and irrigated environments. (JPEG 37 KB) [file 40064_2013_704_MOESM2_ESM.jpeg]
